# Coevolution of the Ile1,016 and Cys1,534 Mutations in the Voltage Gated Sodium Channel Gene of *Aedes aegypti* in Mexico

**DOI:** 10.1371/journal.pntd.0004263

**Published:** 2015-12-11

**Authors:** Farah Z. Vera-Maloof, Karla Saavedra-Rodriguez, Armando E. Elizondo-Quiroga, Saul Lozano-Fuentes, William C. Black IV

**Affiliations:** Department of Microbiology, Immunology and Pathology, Colorado State University, Fort Collins, Colorado, United States of America; Centers for Disease Control and Prevention, UNITED STATES

## Abstract

**Background:**

Worldwide the mosquito *Aedes aegypti* (L.) is the principal urban vector of dengue viruses. Currently 2.5 billion people are at risk for infection and reduction of *Ae*. *aegypti* populations is the most effective means to reduce the risk of transmission. Pyrethroids are used extensively for adult mosquito control, especially during dengue outbreaks. Pyrethroids promote activation and prolong the activation of the voltage gated sodium channel protein (VGSC) by interacting with two distinct pyrethroid receptor sites [1], formed by the interfaces of the transmembrane helix subunit 6 (S6) of domains II and III. Mutations of S6 in domains II and III synergize so that double mutants have higher pyrethroid resistance than mutants in either domain alone. Computer models predict an allosteric interaction between mutations in the two domains. In *Ae*. *aegypti*, a Ile1,016 mutation in the S6 of domain II was discovered in 2006 and found to be associated with pyrethroid resistance in field populations in Mexico. In 2010 a second mutation, Cys1,534 in the S6 of domain III was discovered and also found to be associated with pyrethroid resistance and correlated with the frequency of Ile1,016.

**Methodology/Principal Findings:**

A linkage disequilibrium analysis was performed on Ile1,016 and Cys1,534 in *Ae*. *aegypti* collected in Mexico from 2000–2012 to test for statistical associations between S6 in domains II and III in natural populations. We estimated the frequency of the four dilocus haplotypes in 1,016 and 1,534: Val1,016/Phe1,534 (susceptible), Val1,016/Cys1,534, Ile1,016/Phe1,534, and Ile1,016/Cys1,534 (resistant). The susceptible Val1,016/Phe1,534 haplotype went from near fixation to extinction and the resistant Ile1,016/Cys1,534 haplotype increased in all collections from a frequency close to zero to frequencies ranging from 0.5–0.9. The Val1,016/Cys1,534 haplotype increased in all collections until 2008 after which it began to decline as Ile1,016/Cys1,534 increased. However, the Ile1,016/Phe1,534 haplotype was rarely detected; it reached a frequency of only 0.09 in one collection and subsequently declined.

**Conclusion/Significance:**

Pyrethroid resistance in the *vgsc* gene requires the sequential evolution of two mutations. The Ile1,016/Phe1,534 haplotype appears to have low fitness suggesting that Ile1,016 was unlikely to have evolved independently. Instead the Cys1,534 mutation evolved first but conferred only a low level of resistance. Ile1,016 in S6 of domain II then arose from the Val1,016/Cys1,534 haplotype and was rapidly selected because double mutants confer higher pyrethroid resistance. This pattern suggests that knowledge of the frequencies of mutations in both S6 in domains II and III are important to predict the potential of a population to evolve *kdr*. Susceptible populations with high Val1,016/Cys1,534 frequencies are at high risk for *kdr* evolution, whereas susceptible populations without either mutation are less likely to evolve high levels of *kdr*, at least over a 10 year period.

## Author Summary

Constant use of pyrethroid insecticides has driven mosquito populations to develop resistance. In *Aedes aegypti*, the primary mosquito vector of dengue, yellow Fever, and chikungunya viruses, pyrethroid resistance is primarily associated with mutations in the voltage-gated sodium channel protein. One mutation occurs in codon 1,016 and involves a replacement of valine with isoleucine (Ile1, 016), and a second located in subunit 6 of domain III in codon 1,534, replaces phenylalanine with cysteine (Cys1,534). In Mexico, we found that Cys1,534 was present in the same mosquito collections that were previously analyzed for Ile1,016. In this study, we performed a linkage disequilibrium analysis on both Ile1,016 and Cys1,534 in Mexican collections from 2000–2012. Our analysis suggests that pyrethroid resistance requires the sequential evolution of the two mutations and that Cys1,534 must occur first and appears to enable the Ile1,016 mutation to survive.

## Introduction

Worldwide *Aedes aegypti* (L.) mosquitoes are the principal urban vectors of dengue, chikungunya, and yellow fever viruses. Approximately 2.5 billion people (40% of the human population) currently live with the risk of dengue transmission. In Mexico, *Ae*. *aegypti* is the primary vector of the four dengue virus serotypes (DENV1-4), the causative agents of dengue fever (DF), dengue hemorrhagic fever (DHF) and dengue shock syndrome (DSS). Mexico is severely affected by DF, DSS, and DHF because all four dengue serotypes co-occur in most states of Mexico. A recent review of dengue disease in Mexico [2] reported an increase in incidences from 1.72 per 100,000 in 2000 to 14.12 per 100,000 in 2011.

Currently the most effective means to reduce dengue transmission by *Ae*. *aegypti* is through reduction of larval and adult populations. In Mexico larval reduction is accomplished chiefly through the application of the organophosphate temephos to peridomestic larval breeding sites and through physical source reduction or alteration of potential water-holding containers. Following recommendations of the official Mexican policy for vector control, (NOM-032-SSA2-2002), pyrethroids were almost exclusively used to control adults in and around homes from 1999 to 2010.

Pyrethroid insecticides prolong the opening of the voltage gated sodium channel protein (VGSC) in insect nerves to produce instant paralysis and ‘‘knock-down.” The α-subunit of VGSC has four repeat domains, labeled I-IV, each of which contains six transmembrane helix segments, S1-S6. Pyrethroids preferentially bind to the open state of vgsc by interacting with two distinct receptor sites formed by the interfaces of the transmembrane helix S6 of domains II and III, respectively [1]. The original computer modeling studies [3] suggest that simultaneous binding of pyrethroids to S6 in both domains II and III is necessary to efficiently lock sodium channels in the open state. These models also predict that mutations in the S6 of domain III allosterically alter S6 in domain II via a small shift of IIS6 thus establishing a molecular basis for the coevolution of S6 mutations in domains II and III in conditioning pyrethroid resistance.

In 2006 we described a mutation, Ile1,016, in the S6 of domain II in *Ae*. *aegypti* that is associated with very high knock-down resistance (*kdr*) to the pyrethroid insecticide permethrin in mosquitoes homozygous for this mutation. We examined collections of *Ae*. *aegypti* from Mexico during 1996–2009 [4] and found that the overall Ile1,016 frequency increased from 0.1% in 1996–2000, to 2%–5% in 2003–2006, to 38.3%–88.3% in 2007–2009 depending upon collection location. In 2010 another *vgsc* mutation was described in the S6 of domain III in *Ae*. *aegypti* that was also strongly correlated with *kdr* and involved a cysteine replacement (Cys1,534Phe) [5–7]. A general trend in these studies was that Cys1,534 frequencies were generally higher and increased more rapidly than Ile1,016 frequencies in natural populations.

Based upon these observations and on the dual binding model [3], we analyzed fresly collected DNA from *Ae*. *aegypti* for Ile1,016 and Cys1,534 while DNA previously analyzed for Ile1,016 [4] were tested for the presence of Cys1,534. The purpose of this study was to test the hypothesis that mutations in the S6 of domains II and III coevolve in a dependent manner through various allosteric interactions as suggested by computer models [3, 8]. An analysis of linkage disequilibrium was performed on the two alleles in 1,016 (Val 1,016 (susceptible), Ile 1,016(resistant)) and on the two alleles in 1,534 (Phe 1,534 (susceptible), Cys1,534 (resistant)) to assess whether alleles at 1,534 and 1,016 evolve independently or in a correlated fashion through epistasis.

## Materials and Methods

### Mosquitoes

**Fig 1:**
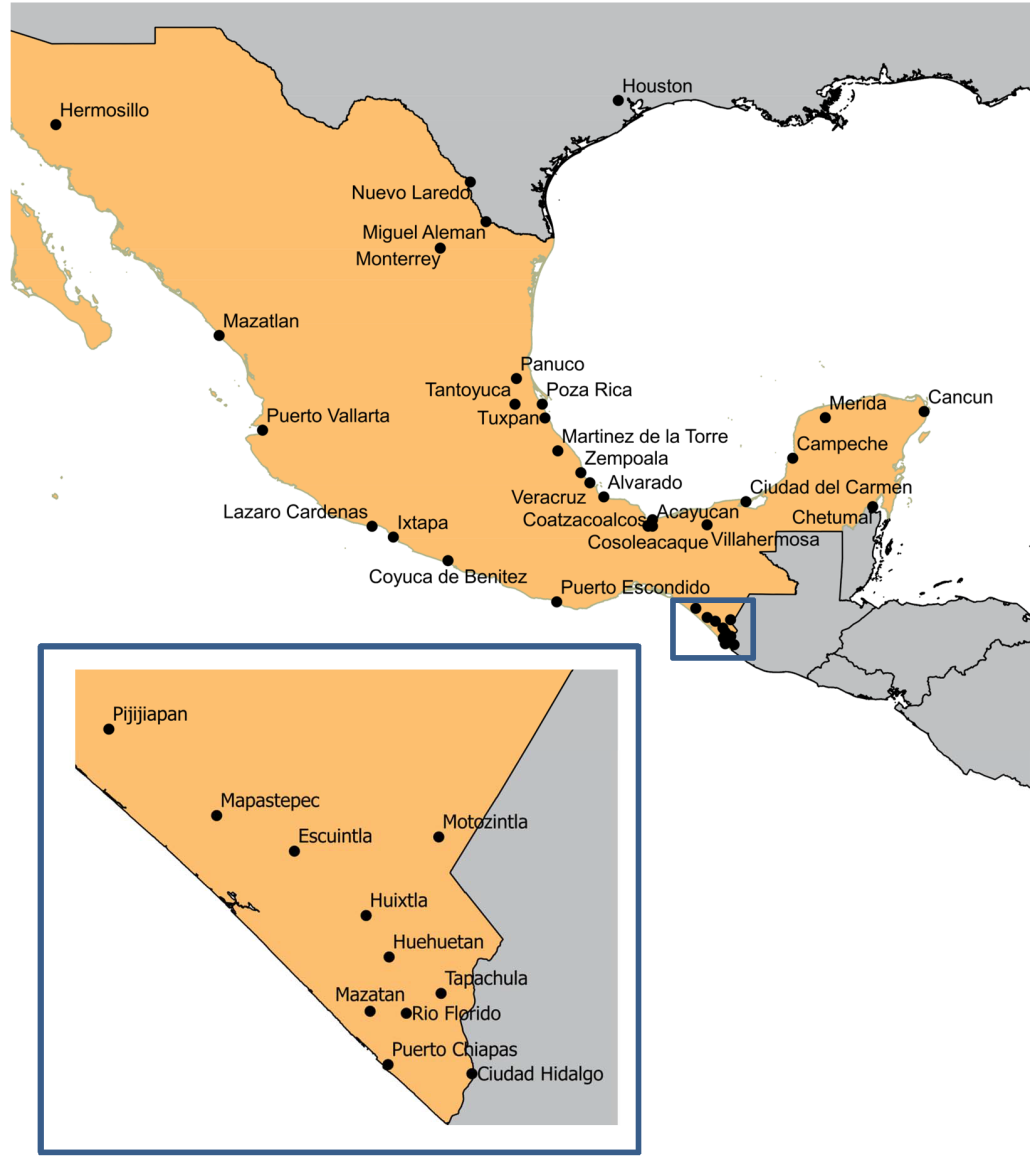
Locations of *Aedes aegypti* collections used in the present study.

Larval mosquitoes were collected from the locations mapped in Fig 1 and listed in Table 1. At each collection site, we collected immatures from at least 30 different containers in each of three different areas located at least 100 m apart. This included water storage containers and discarded trash containers such as plastic pails, tires, and cans. Larvae were returned to the laboratory where they were reared to adults and then identified to species. The Viva Caucel collection was west of the city of Merida in Yucatán State (20.9979639°, 089.7174611°). The Vergel collection was from eastern Merida (Fig 1) (20.9575694°, -89.5886889°). Both were collected in 2011 by Universidad Autónoma de Yucatán. DNA was isolated from individual adult mosquitoes by the salt extraction method [9] and suspended in 150 mL of TE buffer (10 mM Tris-HCl, 1 mM EDTA, pH 8.0). The SNP identification, allele-specific polymerase chain reaction (PCR), melting curve conditions, and genotype readings followed published procedures [6, 10–12].

**Table 1:** Locations, collection years, sample size and Ile1,016 and Cys1,534 genotypes. VV = Val1,016 homozygotes, VI = Val1,016/Ile1,016 heterozygotes, II = Ile1,016 homozygotes, FF = Phe1,534 homozygotes, FC = Phe1,534/Cys1,534 heterozygotes, CC = Cys1,534 homozygotes for *Ae*. *aegypti* in Mexico from 1996 to 2012.

State				VV			VI			II		
	City (Latitude/Longitude)	Year	Sample size	FF	FC	CC	FF	FC	CC	FF	FC	CC
**Texas (U.S.A.)**												
	Houston (29.75944/-95.36193)	1999	47	47	0	0	0	0	0	0	0	0
**Tamaulipas**												
	Nuevo Laredo (27.5/-96.4667)	2000	50	49	0	0	1	0	0	0	0	0
	Miguel Aleman (26.399543/-99.031043)	1999	47	47	0	0	0	0	0	0	0	0
**Nuevo Leon**												
	Monterrey (25.6667/-100.30000)	1999	47	47	0	0	0	0	0	0	0	0
		2008	47	3	7	6	0	9	14	0	1	7
**Veracruz**												
	Panuco (22.05346/-98.18661)	2002	50	26	5	0	0	16	2	0	0	1
	Tuxpan (20.956275/-97.406467)	2012	54	0	0	1	0	0	24	0	0	29
	Tantoyuca (21.34176/-98.22774)	2000	47	47	0	0	0	0	0	0	0	0
		2002	50	50	0	0	0	0	0	0	0	0
		2003	41	40	0	0	1	0	0	0	0	0
		2008	47	3	6	1	0	12	7	0	0	18
	Poza Rica (21.34366/-97.47079)	2000	46	46	0	0	0	0	0	0	0	0
		2002	47	47	0	0	0	0	0	0	0	0
		2003	50	32	16	0	0	1	0	0	0	1
		2008	39	1	2	2	0	15	1	0	0	18
		2012	38	0	0	1	1	1	11	0	0	24
	Martınez de la Torre (20.04999/-97.03883)	2000	47	47	0	0	0	0	0	0	0	0
		2002	47	39	0	0	8	0	0	0	0	0
		2003	30	24	3	0	0	3	0	0	0	0
		2008	48	0	2	7	0	3	21	0	0	15
		2012	54	0	0	0	0	0	15	0	1	38
	Zempoala (19.44489/-92.90000)	2000	47	46	1	0	0	0	0	0	0	0
		2002	47	46	1	0	0	0	0	0	0	0
		2003	30	30	0	0	0	0	0	0	0	0
		2012	54	0	0	4	0	2	17	1	4	26
	Veracruz (19.16508/-96.21625)	2008	46	0	0	7	0	0	22	0	0	17
		2012	54	0	0	0	1	1	18	1	0	33
	Alvarado (18.77422/-95.76356)	2000	47	46	1	0	0	0	0	0	0	0
		2002	50	37	5	0	7	1	0	0	0	0
		2003	49	45	3	1	0	0	0	0	0	0
		2012	54	0	2	6	2	1	30	0	0	13
	Acayucan (17.96196/-94.41255)	2002	47	45	1	0	0	1	0	0	0	0
	Cosoleacaque (17.96196/-94.53605)	2000	47	40	0	0	0	7	0	0	0	0
		2002	47	39	0	0	1	7	0	0	0	0
		2008	47	16	13	4	2	6	3	0	0	3
	Minatitlan (17.97972/-94.54083)	2002	50	44	2	0	0	4	0	0	0	0
		2003	45	43	2	0	0	0	0	0	0	0
	Coatzacoalcos (18.14081/-94.4131)	2002	50	48	0	0	0	2	0	0	0	0
		2003	47	43	2	2	0	0	0	0	0	0
		2008	50	0	0	23	0	0	27	0	0	0
		2012	54	0	0	9	0	1	27	1	0	16
**Tabasco**												
	Villahermosa (18/-92.90000)	2000	47	47	0	0	0	0	0	0	0	0
**Campeche**												
	Ciudad del Carmen (18.641496/-91.82075)	2000	47	47	0	0	0	0	0	0	0	0
	Campeche (19.845446/-90.523673)	2000	47	47	0	0	0	0	0	0	0	0
**Yucatan**												
	Merida (21.0124/-89.63083)	2000	47	47	0	0	0	0	0	0	0	0
		2007	47	6	2	0	1	27	1	0	4	6
	Merida-Center (20.9519/-89.6408)	2000	47	47	0	0	0	0	0	0	0	0
	Merida-East	2000	47	47	0	0	0	0	0	0	0	0
	Merida-North	2000	47	47	0	0	0	0	0	0	0	0
	Merida-South	2000	37	37	0	0	0	0	0	0	0	0
	Merida-West	2000	47	47	0	0	0	0	0	0	0	0
**Quintana Roo**												
	Cancun I (21.14/-86.8800)	2000	47	47	0	0	0	0	0	0	0	0
	Cancun II (21.14/-86.8800)	2000	36	36	0	0	0	0	0	0	0	0
	Chetumal-Calderitas (18.5/-88.30000)	2007	47	18	2	1	0	16	2	0	1	7
	Chetumal-Lagunitas (18.50814/-88.29721)	2007	40	1	0	0	1	5	4	0	1	28
	Chetumal-Lazaro Cardenas	2007	47	1	4	1	2	10	14	0	0	15
	Chetumal-Antorchistas	2008	30	11	1	0	0	15	0	0	0	3
	Chetumal-Solidaridad	2008	47	18	3	0	0	22	2	1	0	1
**Chiapas**												
	Ciudad Hidalgo (14.67902/-92. 15102)	2006	47	32	3	0	0	9	0	0	1	2
		2008	44	2	13	14	3	8	4	0	0	0
	Motozintla (15.37056/-92.24789)	2006	47	46	0	0	1	0	0	0	0	0
		2008	47	24	21	2	0	0	0	0	0	0
	Rio Florido (14.855625/-92.342744)	2006	47	35	1	0	0	4	5	0	0	2
		2008	46	6	16	20	0	1	3	0	0	0
	Puerto Chiapas (14.705707/-92.396214)	2006	48	42	6	0	0	0	0	0	0	0
		2008	47	4	21	22	0	0	0	0	0	0
	Mazatan (14.8615/-92.44862)	2006	47	37	10	0	0	0	0	0	0	0
		2008	47	4	25	9	1	3	5	0	0	0
	Huehuetan (15.01996/-92.39306)	2006	47	47	0	0	0	0	0	0	0	0
		2008	47	5	28	13	0	1	0	0	0	0
	Huixtla (15.14116/-92.46021)	2006	47	42	3	0	2	0	0	0	0	0
		2008	46	4	5	37	0	0	0	0	0	0
	Escuintla (15.32909/-92.66992)	2006	47	11	12	1	0	16	1	0	0	6
		2008	45	0	6	32	0	1	6	0	0	0
	Mapastepec (15.43309/-92.89723)	2006	47	28	18	1	0	0	0	0	0	0
		2008	47	20	20	7	0	0	0	0	0	0
	Pijijiapan (15.68546/-93.21236)	2006	47	26	17	4	0	0	0	0	0	0
		2008	47	4	30	13	0	0	0	0	0	0
	Tapachula I (14.91368/-92.24116)	2000	47	46	1	0	0	0	0	0	0	0
	Tapachula II	2000	37	36	1	0	0	0	0	0	0	0
**Oaxaca**												
	Puerto Escondido (15.865535/-97.069447)	2000	47	47	0	0	0	0	0	0	0	0
**Guerrero**												
	Coyuca de Benitez (17.008464/-100.085473)	2000	47	44	3	0	0	0	0	0	0	0
	Ixtapa (17.660628/-101.601346)	2000	47	47	0	0	0	0	0	0	0	0
**Michoacán**												
	Lazaro Cardenas (17.959826/-102.191412)	2000	47	47	0	0	0	0	0	0	0	0
**Jalisco**												
	Puerto Vallarta (20.622018/-105.228457)	2000	50	50	0	0	0	0	0	0	0	0
**Sinaloa**												
	Mazatlan (23.2467/-106.43318)	2000	47	47	0	0	0	0	0	0	0	0
**Sonora**												
** **	Hermosillo (29.089186/-110.96133)	2000	47	47	0	0	0	0	0	0	0	0
**Total**			4,039									

### Testing for associations between *vgsc* genotypes and kdr phenotypes

The F_3_ generation of the Viva Caucel and Vergel strains were exposed to 25 μg permethrin (Chem Service, West Chester, PA) coated 250 mL Wheaton bottles. In each bottle approximately fifty 3–4 days old mosquitoes were exposed for one hour. Active mosquitoes were transferred to cardboard cups and frozen at -80°C and formed the ‘alive’ group. Knocked down mosquitoes were transferred to a second cardboard cup and placed into an incubator at 28°C and 70% humidity. After four hours, newly recovered mosquitoes were aspirated, frozen and labeled as ‘recovered’. The mosquitoes that remained inactive were scored as ‘dead’.

### Linkage disequilibrium analysis

There are four potential 1,016/1,534 dilocus haplotypes: Val1,016/Phe1,534 (VF), Val1,016/Cys1,534 (VC), Ile1,016/Phe1,534 (IF), Ile1,016/Cys1,534 (IC). The number of times (T_ij_) that an allele at locus *i* = 1,016 appears with an allele at locus *j* = 1,534 was determined by the program LINKDIS [13]. The program then calculated composite disequilibrium frequencies [14] because the phase of alleles at 1,016 and 1,534 are unknown in double heterozygotes. An unbiased estimate of the composite disequilibrium coefficient Δ_ij_ [14, 15] was calculated as:

$$\Delta_{\text{ij}}\;=(\text{N}/(\text{N}-\;1))(({\text{T}}_{\text{ij}}\;/\text{N})-2p_{i}p_{j})$$

Where N is the sample size and *p*_*i*_ and *p*_*j*_ are the frequencies of alleles at locus *i* = 1,016 and locus *j* = 1,534 respectively. Bayesian 95% Highest Density Intervals (HDI) around *p*_*i*_ and *p*_*j*_ were calculated in WinBUGS[16].

A χ^2^ test was performed to determine if significant disequilibrium exists among all alleles at 1,016 and 1,534. The statistic was calculated and summed over all two-allele-interactions [15]:

$$\chi_{\left[1\;d.f.\right]}^{2}=N\sum_{i}\sum_{j}(\frac{\Delta_{ij}^{2}}{p_{i}p_{j}})$$

The linkage disequilibrium correlation coefficient *R*_*ij*_ [15] is distributed from -1 (both mutations *trans*) to 0 (1,534 and 1,016 mutations occur independently), to 1 (both mutations *cis*) and therefore provides a standardized measure of disequilibrium:

$$R_{ij}=\;\Delta_{ij}\;/\;\sqrt{\left({\text{p}}_{i}(1\;-\;{\text{p}}_{i})\;+\;C_{i})\;({\text{p}}_{j}(1\;-\;{\text{p}}_{j})\;+\;C_{j}\right)}$$

Where the *C*_*i*_ term corrects for departures from Hardy-Weinberg expectations:

$$C_{i}=\;H_{obs}(i)-\;p_{i}^{2}$$

where *H*_*obs*_ *(i)* is the observed frequency of *i* homozygotes. Departures from Hardy-Weinberg expectations were also expressed as Wright’s inbreeding coefficient (F_IS_) and calculated as 1- (*H*_*exp*_/2*p* (1- *p*)) where *H*_*exp*_ is the observed frequency of heterozygotes. A χ^2^ test of the hypothesis F_IS_ = 0 with one degree of freedom is:

$$\chi_{\left[1\;d.f.\right]}^{2}=\frac{{N(H_{exp}-H_{obs})}^{2}}{\sum_{i}p_{i}^{2}+\;{\left(\sum_{i}p_{i}^{2}\right)}^{2}-2\;\sum_{i}p_{i}^{3}}$$

## Results

### Testing for associations between *vgsc* genotypes and kdr phenotypes

The locations of all sampling sites are shown in Fig 1 and the latitude and longitude coordinates are provided in Table 1. The sample sizes and numbers of the nine dilocus genotypes (Three 1,534 genotypes x Three 1,016 genotypes) are listed in Table 1. From a total of 615 treated mosquitoes in Viva Caucel, 17.6% (n = 108) were scored as alive, 15.6% (n = 96) as recovered and 66.8% (n = 411) as dead (Table 2). Genotypes at 1,016 and 1,534 were identified in 95 randomly chosen individuals from each group. From a total of 337 treated Vergel mosquitoes, 48.1% (n = 162) were scored as alive, 20.5% (n = 68) as recovered and 31.5% (n = 106) as dead. We randomly chose 95, 68 and 95 Vergel individuals from each group, respectively to obtain the genotypes at 1,016 and 1,534 (Table 2).

**Table 2:** Phenotypes and genotypes at loci 1,016 and 1,534 in Viva Caucel and Vergel.

Site	Genotype 1,016	Genotype 1,534	Alive	Recovered	Dead	Total
**Viva Caucel**						
	AA	GG	91	66	11	168
	AA	GT	0	0	0	0
	AA	TT	0	0	0	0
	AG	GG	0	28	40	68
	AG	GT	3	0	17	20
	AG	TT	0	0	0	0
	GG	GG	0	1	7	8
	GG	GT	0	0	18	18
	GG	TT	1	0	1	2
	Total		95	95	94	284
**Vergel**						
	AA	GG	87	43	26	156
	AA	GT	1	0	0	1
	AA	TT	0	0	0	0
	AG	GG	6	22	68	96
	AG	GT	0	1	0	1
	AG	TT	0	0	0	0
	GG	GG	0	0	1	1
	GG	GT	0	0	0	0
	GG	TT	1	0	1	2
	Total		95	66	96	257

In Viva Caucel, the frequency of the Ile1,106 allele was 0.746 and the frequency of the Cys1,534 allele was 0.926 (Table 3), while in Vergel Ile1,016 was at a slightly higher frequency of 0.80 while the Cys1,534 allele was close to fixation at 0.988. The Ile1,106 and Cys1,534 alleles were in positive disequilibrium in Viva Caucel, but were only marginally significant in Vergel.

**Table 3:** Genotype and allele frequencies at loci 1,016 and 1,534 in Viva Caucel and Vergel.

Locus	Genotype	Viva Caucel	Vergel
**1,016**			
	AA	168	157
	AG	88	97
	GG	28	7
	frequency A =	**0.746**	**0.800**
	frequency G =	0.254	0.200
**1,534**			
	GG	244	253
	GT	38	2
	TT	2	2
	frequency G =	**0.926**	**0.988**
	frequency T =	0.074	0.012

Genotypes at the 1,016 and 1,534 loci were not independent, in agreement with the linkage disequilibrium analysis in Table 4. Table 5 is a three-way contingency analysis of genotypes at loci 1,016, 1,534 and numbers alive or dead individuals in Viva Caucel. Numbers of alive were not independent of genotypes at the 1,016 locus; specifically, numbers of alive were significantly greater in Ile1,016 homozygous mosquitoes than in heterozygotes or in Val1,016 homozygotes. Numbers of alive were also not independent of genotypes at the 1,534 locus; specifically, numbers of alive were significantly greater in Cys1,534 homozygous mosquitoes than in heterozygotes or in Phe1,534 homozygotes. In general, numbers of alive in the Viva Caucel strain were not independent of genotypes at either locus.

**Table 4:** Linkage disequilibrium analysis at loci 1,016 and 1,534 in Viva Caucel and Vergel.

Collection site	Dilocus Genotype	Observed	Expected	R_ij_	χ^2^	prob.
**Viva Caucel**						
	Ile/Phe	10	31.4	-0.6628	124.75	5.76E-29
	Ile/Cys	414	392.6	0.6628		
	Val/Phe	32	10.6	0.6628		
	Val/Cys	112	133.4	-0.6628		
**Vergel**						
	Ile/Phe	1.5	4.8	-0.1132	3.64	0.0565
	Ile/Cys	409.5	406.2	0.1132		
	Val/Phe	4.5	1.2	0.1132		
	Val/Cys	98.5	101.8	-0.1132		

**Table 5:** Three-way tests of independence between numbers alive, recovered or dead and genotypes at *vgsc* loci 1,016 and 1,534.

Site	Hypothesis tested	G	d.f.	Prob.	Hypothesis supported?
**Viva Caucel- No.alive vs dead**					
	1,016 X 1,534 independent?	77.7(33%)	4	5.48E-16	No
	1,016 X No. alive independent?	139.1(60%)	2	6.35E-31	No
	No. alive = in AA(54.2%) vs AG(3.4%)?	(113.80)	(1)	1.44E-26	No
	No. alive = in AA(54.2%) vs GG(3.5%)?	(65.75)	(1)	5.12E-16	No
	No. alive = in AG(3.4%) vs GG(3.5%)?	(0.22)	(1)	6.41E-01	Yes
	1,534 x No. alive independent?	33.02(14%)	2	6.75E-08	No
	No. alive = in GG(38.6%) vs GT(7.9%)?	(4.77)	(1)	2.89E-02	No
	No. alive = in GG(38.6%) vs TT(1/2)?	(0.35)	(1)	5.53E-01	Yes
	No. alive = in GT(7.9%) vs TT(1/2)?	(33.02)	(1)	9.12E-09	No
	1,016 X 1,534 x No. alive interaction	-17.91(-8%)	4	-	-
	1,016 X 1,534 x No. alive independent?	231.83	12	8.32E-43	No
**Vergel- No. alive versus dead**					
	1,016 X 1,534 independent?	27.93(23%)	4	1.29E-05	No
	1,016 X No. alive independent?	88.27(73%)	2	6.81E-20	No
	No. alive = in AA(56%) vs AG(6.2%)?	(88.27)	(1)	5.72E-21	No
	No. alive = in AA(56%) vs GG(1/3)?	(2.83)	(1)	9.25E-02	No
	No. alive = in AG(6.2%) vs GG(1/3)?	(7.66)	(1)	5.63E-03	No
	1,534 x No. alive independent?	0.53(0%)	2	7.69E-01	Yes
	No. alive = in GG(36.8%) vs GT(1/2)?	(0.25)	(1)	6.14E-01	Yes
	No. alive = in GG(36.8%) vs TT(1/2)?	(0.42)	(1)	5.15E-01	Yes
	No. alive = in GT(1/2%) vs TT(1/2)?	(0.00)	(1)	9.61E-01	Yes
	1,016 X 1,534 x No. alive interaction	4.94(4%)	4	2.94E-01	Yes
	1,016 X 1,534 x No. alive independent?	121.66	12	2.88E-20	No
**Viva Caucel- Recovered versus dead**					
	1,016 X 1,534 independent?	56.65(40%)	4	1.47E-11	No
	1,016 X recovery independent?	70.51(49%)	2	4.89E-16	No
	No. recovered = in AA(39.3%) vs AG(31.8%)?	(45.24)	(1)	1.74E-11	No
	No. recovered = in AA(39.3%) vs GG(3.6%)?	(53.26)	(1)	2.92E-13	No
	No. recovered = in AG(31.8%) vs GG(3.6%)?	(6.45)	(1)	1.11E-02	No
	1,534 x recovery independent?	44.20(31%)	2	2.52E-10	No
	No. recovered = in GG(39.8%) vs GT(0%)?	(5.14)	(1)	2.33E-02	No
	No. recovered = in GG(39.8%) vs TT(0/2)?	(0.96)	(1)	3.26E-01	Yes
	No. recovered = in GT(0%) vs TT(0/2)?	(44.04)	(1)	3.21E-11	No
	1,016 X 1,534 x recovery interaction	-27.95(-19%)	4	-	-
	1,016 X 1,534 x recovery independent?	143.40	12	1.23E-24	No
**Vergel- Recovered versus dead**					
	1,016 X 1,534 independent?	17.59(42%)	4	1.49E-03	No
	1,016 X recovery independent?	21.07(50%)	2	2.66E-05	No
	No. recovered = in AA(27.4%) vs AG(23.7%)?	(21.01)	(1)	4.58E-06	No
	No. recovered = in AA(27.4%) vs GG(0/3)?	(1.67)	(1)	1.97E-01	Yes
	No. recovered = in AG(23.7%) vs GG(0/3)?	(0.40)	(1)	5.27E-01	Yes
	1,534 x recovery independent?	0.71(2%)	2	6.99E-01	Yes
	No. recovered = in GG(25.7%) vs GT(1/2)?	(0.29)	(1)	5.92E-01	Yes
	No. recovered = in GG(25.7%) vs TT(0/2)?	(0.01)	(1)	9.21E-01	Yes
	No. recovered = in GT(1/2) vs TT(0/2)?	(0.71)	(1)	3.99E-01	Yes
	1,016 X 1,534 x recovery interaction	2.39(6%)	4	6.64E-01	Yes
	1,016 X 1,534 x mortality independent?	41.76	12	3.65E-05	No

However, a problem with this analysis is that genotypes at the two loci are not independent. In this and previous studies [10, 11], Ile1,016 homozygous mosquitoes have the greatest survival, while few, if any heterozygotes or Val1,016 homozygotes survive. To evaluate Cys1,534 genotypes independently of Ile1,016 homozygous mosquitoes, we only compared the three Cys1,534 genotypes among Ile1,016 heterozygotes and Val1,016 homozygotes. A significantly larger proportion of Cys1,534 homozygotes survived.

Table 5 also shows the contingency analyses of Vergel mosquitoes. Genotypes at the 1,016 and 1,534 loci were not independent, while they were marginally significant in the linkage disequilibrium analysis in Table 4. Numbers of alive were not independent of genotypes at the 1,016 locus again because numbers of alive were significantly greater in Ile1,016 homozygous mosquitoes than in heterozygotes or in Val1,016 homozygotes. Numbers of alive were however independent of genotypes at the 1,534 locus; specifically because Cys1,534 was almost fixed in the Vergel strain.

Table 5 also shows the three-way contingency analysis between genotypes at loci 1,016 and 1,534 and the numbers recovered or dead in Viva Caucel. As in Table 4, genotypes at the 1,016 and 1,534 loci were not independent. The numbers of recovered mosquitoes were not independent of genotypes at the 1,016 locus; specifically-numbers recovered were significantly greater in Ile1,016 homozygous mosquitoes than in heterozygotes or in Val1,016 homozygotes. Numbers of recovered were also not independent of genotypes at the 1,534 locus; specifically, numbers of alive were significantly greater in Cys1,534 homozygous mosquitoes than in heterozygotes or in Phe1,534 homozygotes.

In general, numbers of recovered in the Viva Caucel strain were heavily dependent on genotypes at both loci. An interesting difference between the two loci is that 32% (28/88) of Ile1,016 heterozygotes recovered while only 3.6% (1/28) of Cys1,534 heterozygotes recovered. This difference was significant (χ^2^ = 7.59, df = 1, *p*-value = 0.006).

Table 5 also shows the same analysis of recovery but in Vergel mosquitoes. Genotypes at the 1,016 and 1,534 loci were not independent, while they were marginally significant in the linkage disequilibrium analysis in Table 4. Numbers of recovered were not independent of genotypes at the 1,016 locus, again because numbers of recovered were significantly greater in Ile1,016 homozygous mosquitoes than in heterozygotes or in Val1,016 homozygotes. However, numbers of recovered were independent of genotypes at the 1,534 locus; specifically because Cys1,534 was approaching fixation in the Vergel strain.

### Spatial and temporal analysis of genotype frequencies

Table 6 contains the frequencies of Ile1,016 and Cys1,534 and their Bayesian 95% HDI. F_IS_ was significantly greater than zero (heterozygote deficiency) in two of the 36 collections where Ile1,016 and Val1,016 alleles were segregating. In contrast, a significant heterozygote deficiency occurred in eight of the 53 collections where Cys1,534 and Phe1,534 were segregating and an heterozygote excess occurred in two collections.

**Table 6:** Frequencies of Ile1,016 and Cys1,534 alleles and the 95% Highest Density Intervals around these frequencies. F_IS_ and the associated probabilities from the χ^2^ test to test whether F_IS_ = 0.

State	City	Year	Ile1,016	95% HDI	FIS	Sig.	Cys1,534	95% HDI	FIS	Sig.
**Texas (U.S.A.)**										
	Houston	1999	0.000	(0.007–0.031)			0.000	(0.007–0.031)		
**Tamaulipas**										
	Nuevo Laredo	2000	0.010^a^	(0.014–0.037)			0.000	(0.007–0.029)		
	Miguel Aleman	1999	0.000	(0.007–0.031)			0.000	(0.007–0.031)		
**Nuevo Leon**										
	Monterrey	1999	0.000	(0.007–0.031)			0.000	(0.007–0.031)		
		2008	0.410	(0.096–0.100)	-0.008		0.760	(0.093–0.079)	0.021	
**Veracruz**										
	Panuco	2002	0.200	(0.070–0.085)	-0.125		0.270	(0.080–0.092)	-0.065	
	Tuxpan	2012	0.760	(0.086–0.074)			1.000	(0.027–0.006)		
	Tantoyuca	2000	0.000	(0.007–0.031)			0.000	(0.007–0.031)		
		2002	0.000	(0.007–0.029)			0.000	(0.007–0.029)		
		2003	0.010^a^	(0.017–0.045)			0.000	(0.008–0.035)		
		2008	0.590	(0.100–0.096)	0.167		0.740	(0.093–0.081)	-0.007	
	Poza Rica	2000	0.000	(0.007–0.031)			0.000	(0.007–0.031)		
		2002	0.000	(0.007–0.031)			0.000	(0.007–0.031)		
		2003	0.030	(0.025–0.048)	0.656	***	0.190	(0.069–0.084)	-0.105	
		2008	0.670	(0.108–0.097)	0.082		0.760	(0.102–0.086)	-0.183	
		2012	0.800	(0.098–0.079)	-0.080		0.960	(0.062–0.033)	0.653	***
	Martınez de la Torre	2000	0.000	(0.007–0.031)			0.000	(0.007–0.031)		
		2002	0.090^a^	(0.047–0.068)			0.000	(0.007–0.031)		
		2003	0.050	(0.042–0.077)	-0.053		0.100	(0.061–0.093)	-0.111	
		2008	0.560	(0.099–0.096)	-0.016		0.950	(0.058–0.035)	-0.055	
		2012	0.860	(0.073–0.057)	-0.161		0.990	(0.035–0.013)	-0.009	
	Zempoala	2000	0.000	(0.007–0.031)			0.010	(0.015–0.040)	-0.011	
		2002	0.000	(0.007–0.031)			0.010	(0.015–0.040)	-0.011	
		2003	0.000	(0.011–0.047)			0.000	(0.011–0.047)		
		2012	0.750	(0.086–0.075)	0.062		0.930	(0.059–0.041)	0.190	
	Veracruz	2008	0.610	(0.101–0.095)			1.000	(0.032–0.007)		
		2012	0.810	(0.080–0.066)	-0.227		0.950	(0.052–0.031)	0.790	***
	Alvarado	2000	0.000	(0.007–0.031)			0.010	(0.015–0.040)	-0.011	
		2002	0.080^a^	(0.066–0.080)	-0.087		0.060	(0.037–0.059)	-0.064	
		2003	0.000	(0.007–0.030)			0.050	(0.034–0.057)	0.368	*
		2012	0.550	(0.094–0.091)	-0.233		0.940	(0.058–0.038)	0.542	***
	Acayucan	2002	0.010	(0.015–0.040)	-0.011		0.020	(0.021–0.046)	-0.022	
	Cosoleacaque	2000	0.070	(0.043–0.065)	-0.080		0.070	(0.043–0.065)	-0.080	
		2002	0.090^a^	(0.047–0.068)	-0.093		0.070	(0.043–0.065)	-0.080	
		2008	0.180	(0.069–0.086)	0.210		0.410	(0.095–0.100)	0.167	
	Minatitlan	2002	0.040	(0.030–0.052)	-0.042		0.060	(0.037–0.059)	-0.064	
		2003	0.000	(0.007–0.032)			0.020	(0.022–0.048)	-0.023	
	Coatzacoalcos	2002	0.020	(0.020–0.043)	-0.020		0.020	(0.020–0.043)	-0.020	
		2003	0.000	(0.007–0.031)			0.060	(0.040–0.062)	0.644	***
		2008	0.270	(0.080–0.092)	-0.370		1.000	(0.029–0.007)		
		2012	0.570	(0.094–0.090)	-0.060		0.970	(0.045–0.024)	0.657	***
**Tabasco**										
	Villahermosa	2000	0.000	(0.007–0.031)			0.000	(0.007–0.031)		
**Campeche**										
	Ciudad del Carmen	2000	0.000	(0.007–0.031)			0.000	(0.007–0.031)		
	Campeche	2000	0.000	(0.007–0.031)			0.000	(0.007–0.031)		
**Yucatan**										
	Merida	2000	0.000	(0.007–0.031)			0.000	(0.007–0.031)		
		2007	0.52^a^	(0.100–0.099)	-0.236		0.500	(0.099–0.099)	-0.404	**
	Merida-Center	2000	0.000	(0.007–0.031)			0.000	(0.007–0.031)		
	Merida-East	2000	0.000	(0.007–0.031)			0.000	(0.007–0.031)		
	Merida-North	2000	0.000	(0.007–0.031)			0.000	(0.007–0.031)		
	Merida-South	2000	0.000	(0.009–0.039)			0.000	(0.009–0.039)		
	Merida-West	2000	0.000	(0.007–0.031)			0.000	(0.007–0.031)		
**Quintana Roo**										
	Cancun I	2000	0.000	(0.007–0.031)			0.000	(0.007–0.031)		
	Cancun II	2000	0.000	(0.009–0.040)			0.000	(0.009–0.040)		
	Chetumal-Calderitas	2007	0.360	(0.092–0.099)	0.170		0.410	(0.096–0.100)	0.167	
	Chetumal-Lagunitas	2007	0.850	(0.089–0.067)	0.020		0.880	(0.084–0.061)	0.314	*
	Chetumal-L. Cardenas	2007	0.600	(0.100–0.095)	-0.148		0.790	(0.089–0.075)	0.111	
	Chetumal-Antorchistas	2008	0.350	(0.112–0.124)	-0.099		0.370	(0.113–0.124)	-0.148	
	Chetumal-Solidaridad	2008	0.300	(0.086–0.096)	-0.221		0.330	(0.089–0.098)	-0.203	
**Chiapas**										
	Ciudad Hidalgo	2006	0.160	(0.065–0.083)	0.286	*	0.180	(0.069–0.086)	0.066	
		2008	0.170	(0.069–0.087)	-0.205		0.650	(0.102–0.094)	-0.046	
	Motozintla	2006	0.010	(0.015–0.040)			0.000	(0.007–0.031)		
		2008	0.000	(0.007–0.031)			0.270	(0.082–0.094)	-0.144	
	Rio Florido	2006	0.140	(0.060–0.079)	0.197		0.200	(0.073–0.088)	0.670	***
		2008	0.040	(0.032–0.056)	-0.045		0.680	(0.098–0.089)	0.144	
	Puerto Chiapas	2006	0.000	(0.007–0.030)			0.060	(0.039–0.061)	-0.067	
		2008	0.000	(0.007–0.031)			0.690	(0.097–0.087)	-0.047	
	Mazatan	2006	0.000	(0.007–0.031)			0.110	(0.052–0.073)	-0.119	
		2008	0.100	(0.050–0.071)	-0.106		0.600	(0.100–0.095)	-0.237	
	Huehuetan	2006	0.000	(0.007–0.031)			0.000	(0.007–0.031)		
		2008	0.010	(0.015–0.040)	-0.011		0.590	(0.100–0.096)	-0.271	
	Huixtla	2006	0.020	(0.021–0.046)	-0.022		0.030	(0.027–0.051)	-0.033	
		2008	0.000	(0.007–0.031)			0.860	(0.081–0.062)	0.552	
	Escuintla	2006	0.310	(0.087–0.097)	0.152		0.470	(0.098–0.100)	-0.196	
		2008	0.080	(0.045–0.068)	-0.084		0.920	(0.068–0.045)	-0.084	
	Mapastepec	2006	0.000	(0.007–0.031)			0.210	(0.074–0.089)	-0.143	
		2008	0.000	(0.007–0.031)			0.360	(0.092–0.099)	0.078	
	Pijijiapan	2006	0.000	(0.007–0.031)			0.270	(0.082–0.094)	0.074	
		2008	0.000	(0.007–0.031)			0.600	(0.100–0.095)	-0.325	*
	Tapachula I	2000	0.000	(0.007–0.031)			0.010	(0.015–0.040)	-0.011	
	Tapachula II	2000	0.000	(0.009–0.039)			0.010	(0.019–0.050)	-0.014	
**Oaxaca**										
	Puerto Escondido	2000	0.000	(0.007–0.031)			0.000	(0.007–0.031)		
**Guerrero**										
	Coyuca de Benitez	2000	0.000	(0.007–0.031)			0.030	(0.027–0.051)	-0.033	
	Ixtapa	2000	0.000	(0.007–0.031)			0.000	(0.007–0.031)		
**Michoacan**										
	Lazaro Cardenas	2000	0.000	(0.007–0.031)			0.000	(0.007–0.031)		
**Jalisco**										
	Puerto Vallarta	2000	0.000	(0.007–0.029)			0.000	(0.007–0.029)		
**Sinaloa**										
	Mazatlan	2000	0.000	(0.007–0.031)			0.000	(0.007–0.031)		
**Sonora**										
	Hermosillo	2000	0.000	(0.007–0.031)			0.000	(0.007–0.031)		

**Fig 2:**
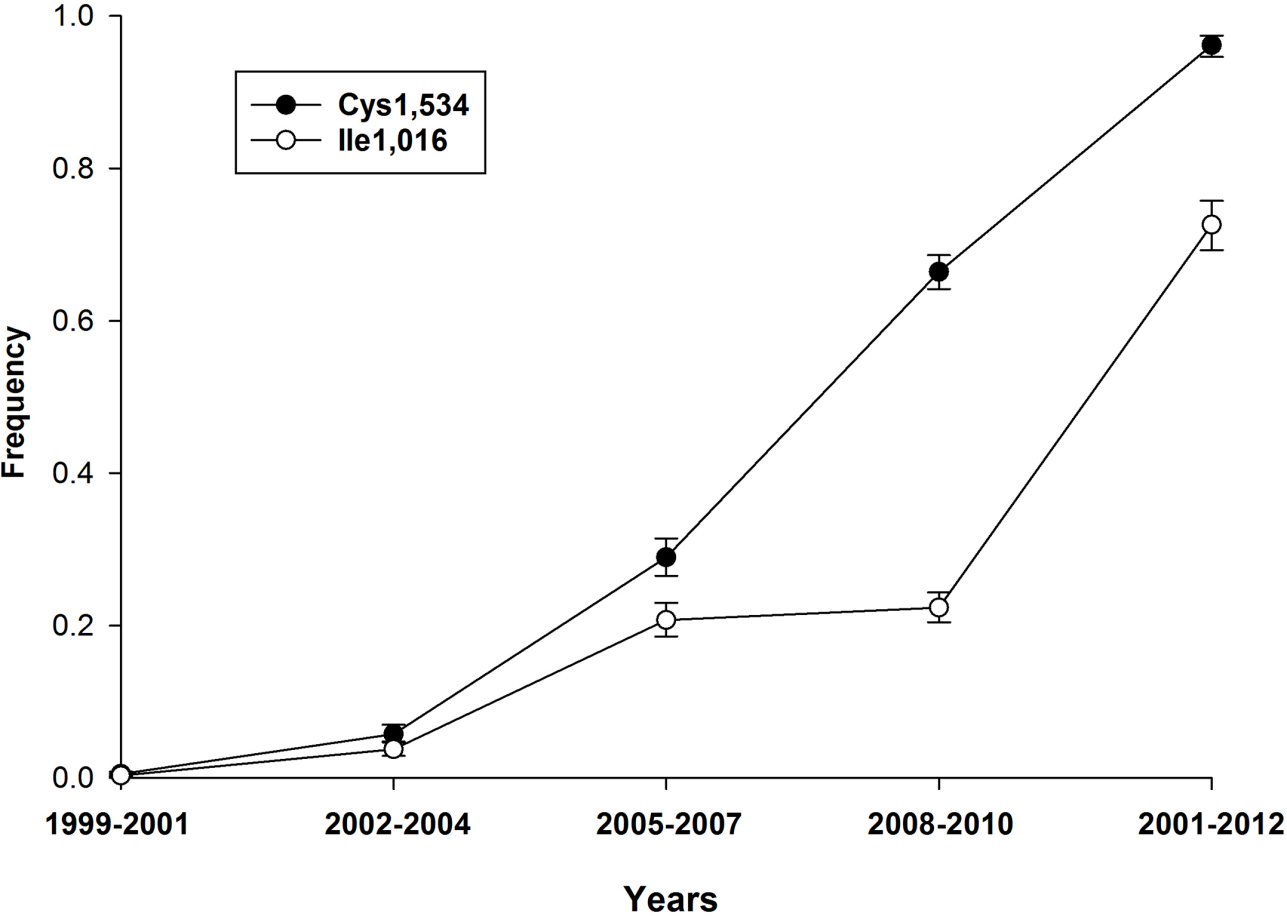
Frequencies of Ile1,016 and Cys1,534 alleles from 1999 to 2012 and their Bayesian 95% HDI.

**Fig 3:**
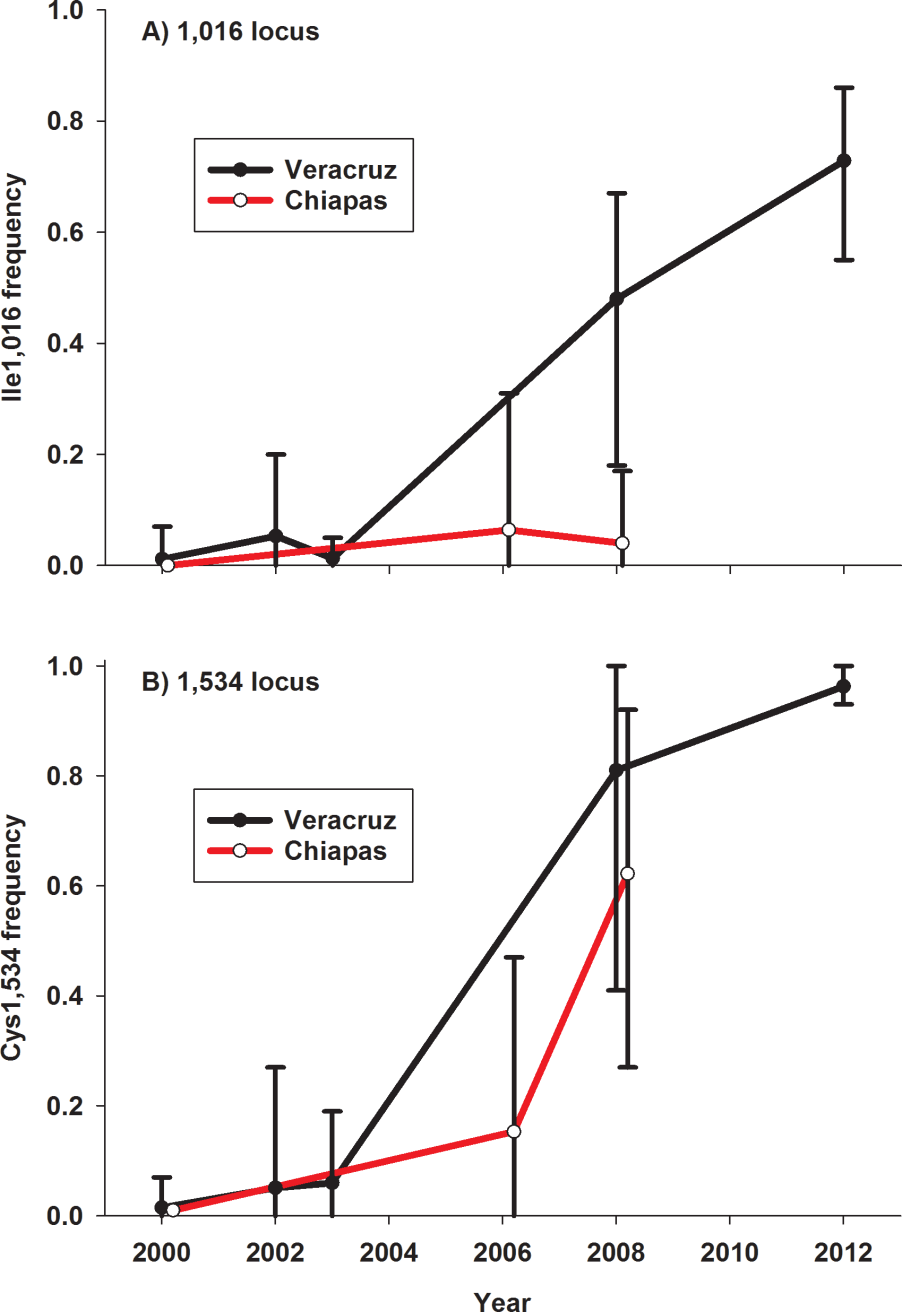
Frequencies of (A) Ile1,016 and (B) Cys1,534 alleles from 2000 to 2012 in cities in Veracruz and Chiapas and their maximum and minimum frequencies among collections in each year.

The frequencies of the Ile1,016 and Cys1,534 alleles from 1999 to 2012 are plotted in Fig 2. The Cys1,534 allele appears sooner and increases more rapidly than Ile1,016. Only the states of Veracruz and Chiapas had sufficient samples over the years to compare the spatial distributions of Ile1,016 and Cys1,534 (Fig 3). It is very clear that Ile1,016 and Cys1,534 were increasing in frequency much earlier in Veracruz state in eastern Mexico than in Chiapas state in southwestern Mexico. It is also clear that in both states Cys1,534 was increasing in frequency much earlier than in Ile1,016. Starting in 2002, the frequency of Cys1,534 was greater than or equal to that of Ile1,016. In a yearly comparison of *Ae*. *aegypti* collection sites, 80 out of 87 sites (Table 6) had a frequency of Cys 1,534 being greater than the frequency of Ile1,016. In 6 of the 7 cases where the frequency of Ile1,016 exceeded that of Cys1,534, the difference was only from 1–2% and values were not different (overlapping 95% HDI). Only in Martınez de la Torre in 2002 was there a credible difference of 9%.

### Linkage disequilibrium analysis

Linkage disequilibrium analysis can only be performed in datasets where alleles are segregating at both loci. There were 34 datasets that met this criteria of the 87 collections listed in Table 1. Table 7 lists the state, city and year of the 34 datasets along with linkage disequilibrium correlation coefficient *R*_*ij*_ and its associated χ^2^ values and the probability of a greater χ^2^_._ Ile1,016 and Cys1,534 were in disequilibrium in the majority (21/34 = 62%) of datasets. For the most part, alleles in 1,534 and 1,016 were evolving in a correlated, dependent fashion. However, this analysis does not provide specific information about the four haplotypes.

**Table 7:** Linkage disequilibrium between Ile1,016 and Cys1,534 mutations in *Aedes aegypti* in Mexican populations.

State	City	Year	R_ij_	χ^2^	Prob
**Nuevo Leon**					
	Monterrey	2008	0.412	8.09	0.004
**Veracruz**					
	Panuco	2002	0.839	28.76	0.000
	Tantoyuca	2008	0.756	31.13	0.000
	Poza Rica	2003	0.483	17.32	0.000
		2008	0.716	17.61	0.000
		2012	0.257	3.81	0.051
	Martınez de la Torre	2003	0.690	12.02	0.001
		2008	0.263	3.08	0.079
		2012	0.087	0.34	0.560
	Zempoala	2012	0.148	1.49	0.222
	Veracruz	2012	0.113	0.95	0.330
	Alvarado	2002	0.007	0.00	1.000
		2012	0.187	2.24	0.134
	Acayucan	2002	0.715	23.23	0.000
	Cosoleacaque	2000	1.000	41.49	0.000
		2002	0.944	34.91	0.000
		2008	0.477	15.11	0.000
	Minatitlan	2002	0.815	29.78	0.000
	Coatzacoalcos	2002	1.000	49.96	0.000
		2012	0.145	1.76	0.185
**Yucatan**					
	Merida	2007	0.774	12.82	0.000
**Quintana Roo**					
	Chetumal-Calderitas	2007	0.856	47.03	0.000
	Chetumal-Lagunitas	2007	0.750	30.17	0.000
	Chetumal-Lazaro Cardenas	2007	0.571	16.4	0.000
	Chetumal-Antorchistas	2008	0.993	22.71	0.000
	Chetumal-Solidaridad	2008	0.740	15.97	0.000
**Chiapas**					
	Ciudad Hidalgo	2006	0.894	51.5	0.000
	Rio Florido	2006	0.929	81.01	0.000
		2008	0.171	1.46	0.227
	Mazatan	2008	0.207	1.38	0.240
	Huehuetan	2008	0.043	0.06	0.806
	Huixtla	2006	0.056	0.14	0.708
	Escuintla	2006	0.728	23.06	0.000
		2008	0.015	0.01	0.920

**Fig 4:**
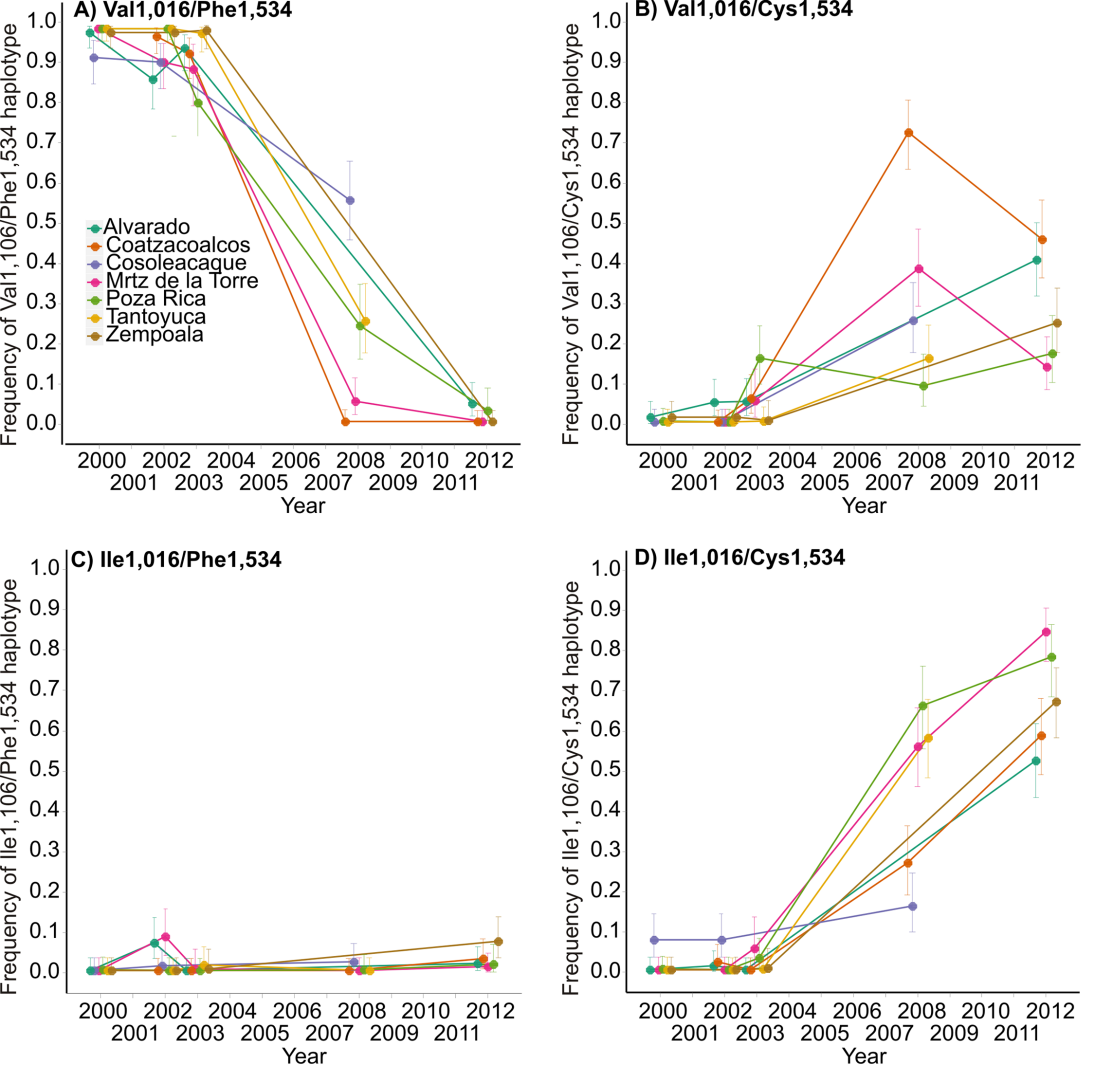
Frequencies of the four potential dilocus haplotypes plotted by year. A) Frequency of the susceptible Val1,016/ Phe1,534 (VF) haplotype, B) Frequency of the Val1,016/Cys1,534 (VC) haplotype, C) Frequency of the Ile1,016/Phe1,534 haplotype and D) Frequency of the resistant Ile1,016/ Cys1,534 (IC) haplotype.

The frequencies of the four potential dilocus haplotypes are plotted by year in Fig 4. The frequency of the susceptible Val1,016/Phe1,534 (VF) haplotype remained high from 1999–2003 (Fig 4A). No collections were made again until 2008, by which time frequencies had dropped to 0–0.6. Four years later, VF was approaching extinction in all collections. Fig 4B plots the frequency of the Val1,016/Cys1,534 (VC) haplotype. From 1999–2003, VC frequencies remained low (0–0.10). By 2008, frequencies had increased to 0.1–0.75. Four years later, VC was declining in frequency in two collections and was increasing in four collections. A very different trajectory occurred for Ile1,016/Phe1,534 (IF) (Fig 4C). From 1999–2002, the IF frequency remained low and only reached as high as 0.1 in two collections. By 2008 frequencies were approaching extinction and four years later similar trends were seen, even though VC and IC frequencies had increased dramatically. Fig 4D is a plot of the frequency of the resistant Ile1,016/Cys1,534 (IC) haplotype. From 1999–2002, the IC frequency was low and only reached 0.1 in one collection. By 2008 frequencies had increased dramatically in all collections and continued to increase in all collections up to 2012 when frequencies ranged from 0.5–0.9.

## Discussion

The frequency of Cys1,534 has increased dramatically in the last decade in several states in Mexico including Nuevo Leon in the north, Veracruz on the central Atlantic Coast, and Chiapas, Quintana Roo and Yucatan in the south. The linkage disequilibrium analysis on the Ile1,016 and Cys1,534 alleles in *Ae*. *aegypti* collected in Mexico from 2000–2012 (Table 7) strongly supports statistical associations between 1,534 and 1,016 mutations in natural populations. Furthermore, the dynamics of haplotype frequencies during that time suggest pyrethroid resistance in the *vgsc* gene requires the sequential evolution of 1,534 and 1,016 mutations. Fig 4C suggests that the Ile1,016/Phe1,534 haplotype has a low fitness, even when pyrethroids are being released. For this reason Ile1,016 is unlikely to have evolved independently. Instead it is much more likely that the Cys1,534 mutation evolved first but conferred only a low level of resistance. This conjecture is strongly supported by the fact that in 80 of 87 collections (92%), the frequency of Cys1,534 was greater than the frequency of Ile1,016.

The findings of this study are different in many respects from those in a study of a Tyr1,575 substitution in *Anopheles gambiae* that occurs just beyond the S6 of domain III, within the linker between domains III and IV [17]. This linker contains a sequence of three amino acids (IFM) that close the sodium channel pore following activation, block the influx of sodium into the cell and restore the membrane resting potential. In contrast, Cys1,534 in *Ae*. *aegypti* occurs in the S6 of domain III. This is close to a Met1,524Ile substitution that has been associated with knockdown resistance in *Drosophila melanogaster* [18] and a Phe1,538Ile mutation that reduces sensitivity to deltamethrin in arthropods and mammals [19, 20].

Mutations in S6 of domain II, such as Phe1,014, Ser1,014 in *An*. *gambie* and Ile1,016 and Gly1,016 in *Ae*. *aegypti* are not directly in the binding pocket, but affect the resistance phenotype by preventing binding of insecticides and changing the conformation of the VGSC [3, 21]. In contrast, a binding site located in a hydrophobic cavity delimited by the IIS4-S5 linker and the IIS5/IIIS6 helices has recently been proposed [22] that it is accessible to the lipid bilayer and lipid-soluble insecticides. The methyl-cyclopropane (or equivalent structure) of pyrethroids and the trichloromethyl group of DDT appear to be critical features for the action of both pyrethroids and DDT. Both insecticides fit into a slot in a small pocket in the main hydrophobic cavity, flanked by Val1,529 and Phe1,530 on IIIS6. The binding site is formed upon opening of the sodium channel and is consistent with observations that pyrethroids bind preferentially to open channels. This binding pocket includes several known mutations in the S6 of domain III that reduce sensitivity to pyrethroids. Two nearby residues (Gly1,535 and Phe1,538) have been previously implicated in resistance in other insect species (23).

Study in which *An*. *gambiae* mosquitos were collected from a range of approximately 2000 km throughout West/Central Africa and had Tyr1,575 occurring at frequencies up to 30% in both M and S forms. Even though this mutation is seen over a large range of the continent, only a single Tyr1,575 haplotype occurred with a Phe1,014 haplotype background (possibly analogous in function to Ile,1016), which infers strong positive selection acting on a recent mutant [17]. In contrast to the present study, Phe1,014 is almost fixed in West Africa and the Tyr1,575 allele is increasing in frequency in M form but not in S form. Thus in contrast to the apparent evolution of Ile1,016 on a Cys1,534 background as reported here in *An*. *gambiae*, Tyr1,575 appears to have evolved on a Phe1,014 background. There are many potential reasons for this difference including the possibility that mutations within the S6 of domain III may produce a different resistance mechanism and have a different impact on fitness than mutations in the linker between domains III and IV. It is also possible that the specific changes of amino acids at these sites are unique and may confer different resistance phenotypes. In either case it seems likely that one of the mutations compensates for deleterious fitness effects of the other mutation and/or confers additional resistance to insecticides.

An interesting difference between the two mutations in the present study is that 32% of Ile1,016 heterozygotes recover from pyrethroid exposure but only 3.6% of Cys1,534 heterozygotes recover. Thus while Cys1,534 in synergy with Ile1,016 may confer greater survival following pyrethroid exposure, Ile1,016 may confer a greater ability to recover following knockdown in heterozygotes.

There was evidence of heterozygote deficiency in eight of the 53 collections and the average F_IS_ among these eight collections was large and positive (0.580) while the average among all collections was 0.052. This suggests that the fitness of Phe1,534 and Cys1,534 homozygotes may be greater than the fitness of G/T heterozygotes (i.e. underdominance). While these parameters have been estimated at the 1,016 locus [23], no similar studies have involved the 1,534 locus and so the stability point beyond which the frequency of either allele would increase has not been determined. Since the Cys1,534 confers some degree of pyrethroid resistance (Tables 2–5), directional selection could increase the frequency of Cys1,534 beyond the underdominance stability point, at which stage the frequency of Cys1,534 would rapidly increase towards fixation.

Little is known of other mutations in the *Ae*. *aegypti vgsc* that may affect pyrethroid resistance. Codon 989 in the “super-kdr” region of domain II was assessed and no mutations were found [11]. Ile, Met and Val alleles occur at codon 1,011 [11] but these alleles were not associated with resistance in our initial survey of 1,318 mosquitoes from the 32 strains throughout Latin America [11]. The recombination dynamics of the *Ae*. *aegypti vgsc* are also poorly understood. Analysis of segregation between alleles at the 1,011 and 1,016 codons in F_3_ showed a high rate of recombination even though the two codons are only separated by a approximately 250 bp intron [11]. A maximum parsimony phylogeny of the intron spanning exons 20 and 21 in 88 mosquitoes with different genotypes in exons 1,011 and 1,016 indicated the presence of three clades with bootstrap support > 80%. These were arbitrarily labelled clades 1–3. The frequencies of Ile1,011, Met1,011, Val1,011, Val1,016, Ile1,016 and Gly1,016 (from Thailand only) were then compared among the three clades. The frequency of Ile1,011 was distributed independently among the three clades, as was Val1,011 and Met1,011. However, there was a very evident excess of Val1,016 alleles in clade 1 and an excess of Ile1,016 alleles in clade 2. Ile1,016 alleles occurred in disequilibrium with a large number of segregating sites in the intron and a large excess of Ile1,016 alleles were found to be associated with clade 2 in the phylogenetic analysis. This pattern is consistent with a hypothesis that a genetic sweep of the Ile1,016 allele and proximate intron sequences has occurred through DDT exposure and subsequently pyrethroid selection. Furthermore, the genetic sweep was recent enough that there has been insufficient time for recombination to disrupt the disequilibrium between the Ile1,016 allele and proximate intron sequences.

Recent work on the dual binding model may shed some light on the next steps in the evolution of pyrethroid resistance in the *vgsc* [8]. The Tyr1,575 mutation in *An*. *gambiae* was introduced alone into an *Ae*.*aegypti* sodium channel (AaNav1-1) [8] and then in combination with Phe1,014. Both substitutions were then functionally examined in *Xenopus* oocytes [8]. Tyr1,575 alone did not alter AaNav1-1 sensitivity to pyrethroids. However, the Tyr1575- Phe1014 double mutant was more resistant to pyrethroids than the Phe1014 mutant channel alone. Further mutational analysis showed that Tyr1,575 could also synergize the effect of Ser1,014 and Trp1,014, but not Gly1,014, or other pyrethroid-resistant mutations in subunit 6 of domains I or II. Computer modeling predicted that Tyr1,575 allosterically alters pyrethroid binding via a small shift of the subunit 6 of domain II. This establishes a molecular basis for the coexistence of Tyr1,575 with Phe1,014 in pyrethroid resistance, and suggests an allosteric interaction between IIS6 and Loop III/IV in the sodium channel.

The rapid increase in Cys1,534 (Fig 4B and 4D) cannot be the result of neutral forces such as genetic drift or founder’s effects. Parallel increases in Cys1,534 frequency occurred throughout Mexico. Even though the forces that caused an increase in the frequency of Cys1,534 are unclear, our results suggest that Ile1,016 in domain IIS6 arose from a Val1,016/Cys1,534 haplotype and was rapidly selected possibly because double mutants confer higher pyrethroid resistance. When combined with Phe1014, the Tyr1,575 mutation in *An*. *gambiae* increased resistance to permethrin and deltamethrin by 9.8- and 3.4-fold, respectively [8].

**Fig 5:**
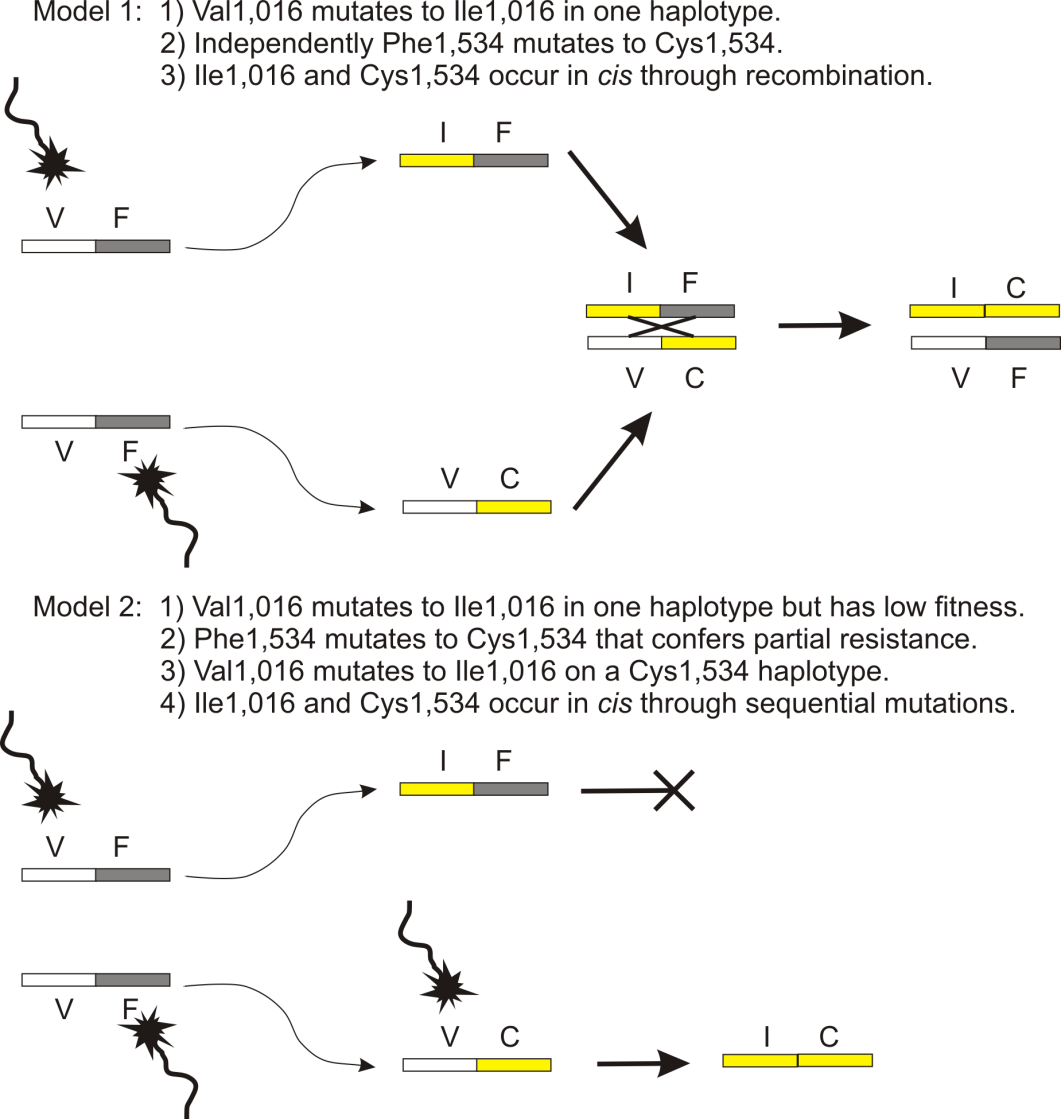
Two models for the evolution of mutations in subunit 6 of domains II and III. Model 1 proposes that the 1,532 and 1,016 mutations occurred independently and became *cis* through crossing over. Model 2 instead proposes that 1,532 mutations occur first because 1,016 mutations confer low fitness. Ile1,016 mutations then arise on a Val1,016/Cys1,534 background.

Fig 5 illustrates two models for the evolution of 1,534 and 1,016 mutations. Model 1 proposes that the 1,534 and 1,016 mutations occurred independently and became *cis* by crossing over. Model 2 instead proposes that 1,534 mutations occurred first because 1,016 mutations confer low fitness. Ile1,016 mutations then arose on a Val1,016/Cys1,534 background. These results suggest that knowledge of the frequencies of both 1,534 and 1,016 mutations are important to predict the potential of a population to evolve *kdr*. Obviously, the frequency of Ile1,016 by itself is a poor predictor (Fig 4C). Populations that are pyrethroid susceptible, but have high Val1,016/Cys1,534 frequencies, are at high risk for rapid *kdr* evolution. If our experience in tracking the frequencies of Ile 1,016 and Cys1,534 mutations over the past 15 years can be extended to other *Ae*. *aegypti* populations, then populations with intermediate to high frequencies of Cys1,534 might only be susceptible for 5–10 years. Conversely, pyrethroid susceptible populations without either mutation are unlikely to develop *kdr* quickly and might be susceptible for up to 10–15 years.

## References

[1] Du YZ, Nomura Y, Satar G, Hu ZN, Nauen R, He SY, et al Molecular evidence for dual pyrethroid-receptor sites on a mosquito sodium channel. P Natl Acad Sci USA. 2013;110(29):11785–90. 10.1073/pnas.1305118110 PMC3718148 23821746

[2] Dantes HG, Farfan-Ale JA, Sarti E. Epidemiological Trends of Dengue Disease in Mexico (2000–2011): A Systematic Literature Search and Analysis. Plos Neglect Trop D. 2014;8(11). 10.1371/journal.pntd.0003158 PMC4222737 25375162

[3] O'Reilly AO, Khambay BPS, Williamson MS, Field LM, Wallace BA, Davies TGE. Modelling insecticide-binding sites in the voltage-gated sodium channel. Biochem J. 2006;396:255–63. 16475981 10.1042/BJ20051925 PMC1462714

[4] Garcia GP, Flores AE, Fernandez-Salas I, Saavedra-Rodriguez K, Reyes-Solis G, Lozano-Fuentes S, et al Recent Rapid Rise of a Permethrin Knock Down Resistance Allele in *Aedes aegypti* in Mexico. Plos Neglect Trop D. 2009;3(10). 10.1371/journal.pntd.0000531 PMC2759509 19829709

[5] Harris AF, Rajatileka S, Ranson H. Pyrethroid Resistance in *Aedes aegypti* from Grand Cayman. Am J Trop Med Hyg. 2010;83(2):277–84. 10.4269/ajtmh.2010.09-0623 20682868 PMC2911171

[6] Yanola J, Somboon P, Walton C, Nachaiwieng W, Somwang P, Prapanthadara LA. High-throughput assays for detection of the F1534C mutation in the voltage-gated sodium channel gene in permethrin-resistant *Aedes aegypti* and the distribution of this mutation throughout Thailand. Tropical Medicine & International Health. 2011;16(4):501–9. 21342372 10.1111/j.1365-3156.2011.02725.x

[7] Alvarez LC, Ponce G, Saavedra-Rodriguez K, Lopez B, Flores AE. Frequency of V1016I and F1534C mutations in the voltage-gated sodium channel gene in *Aedes aegypti* in Venezuela. Pest Manag Sci. 2015;71(6):863–9. 10.1002/ps.3846 24935645

[8] Wang LX, Nomura Y, Du YZ, Liu NN, Zhorov BS, Dong K. A Mutation in the Intracellular Loop III/IV of Mosquito Sodium Channel Synergizes the Effect of Mutations in Helix IIS6 on Pyrethroid Resistance. Mol Pharmacol. 2015;87(3):421–9. 10.1124/mol.114.094730 25523031 PMC4352587

[9] Black WC, DuTeau NM. RAPD-PCR and SSCP analysis for insect population genetic studies In: Crampton J BC, Louis C, editor. The Molecular Biology of Insect Disease Vectors: A Methods Manual. New York: Chapman and Hall 1997 p. 361–73.

[10] Saavedra-Rodriguez K, Strode C, Suarez AF, Salas IF, Ranson H, Hemingway J, et al Quantitative Trait Loci Mapping of Genome Regions Controlling Permethrin Resistance in the Mosquito *Aedes aegypti* . Genetics. 2008;180(2):1137–52. 10.1534/genetics.108.087924 18723882 PMC2567363

[11] Saavedra-Rodriguez K, Urdaneta-Marquez L, Rajatileka S, Moulton M, Flores AE, Fernandez-Salas I, et al A mutation in the voltage-gated sodium channel gene associated with pyrethroid resistance in Latin American *Aedes aegypti* . Insect Mol Biol. 2007;16(6):785–98. 18093007 10.1111/j.1365-2583.2007.00774.x

[12] Urdaneta-Marquez L, Bosio C, Herrera F, Rubio-Palis Y, Salasek M, Black WC. Genetic relationships among *Aedes aegypti* collections in Venezuela as determined by mitochondrial DNA variation and nuclear single nucleotide polymorphisms. Am J Trop Med Hyg. 2008;78(3):479–91. 18337347

[13] Black WC, Krafsur ES. A Fortran Program for the Calculation and Analysis of 2-Locus Linkage Disequilibrium Coefficients. Theor Appl Genet. 1985;70(5):491–6. 10.1007/BF00305981 24253058

[14] Cockerham CC, Weir BS. Digenic Descent Measures for Finite Populations. Genet Res. 1977;30(2):121–47.

[15] Weir BS. Inferences About Linkage Disequilibrium. Biometrics. 1979;35(1):235–54. 497335

[16] Lunn DJ, Thomas A, Best N, Spiegelhalter D. WinBUGS—A Bayesian modelling framework: Concepts, structure, and extensibility. Stat Comput. 2000;10(4):325–37.

[17] Jones CM, Liyanapathirana M, Agossa FR, Weetman D, Ranson H, Donnelly MJ, et al Footprints of positive selection associated with a mutation (N1575Y) in the voltage-gated sodium channel of *Anopheles gambiae* . P Natl Acad Sci USA. 2012;109(17):6614–9. 10.1073/pnas.1201475109 PMC3340067 22493253

[18] Pittendrigh B, Reenan R, ffrenchConstant RH, Ganetzky B. Point mutations in the *Drosophila* sodium channel gene para associated with resistance to DDT and pyrethroid insecticides. Mol Gen Genet. 1997;256(6):602–10. 9435785 10.1007/s004380050608

[19] He HQ, Chen AC, Davey RB, Ivie GW, George JE. Identification of a point mutation in the para-type sodium channel gene from a pyrethroid-resistant cattle tick. Biochem Bioph Res Co. 1999;261(3):558–61. 10.1006/bbrc.1999.1076 10441465

[20] Wang SY, Barile M, Wang GK. A phenylalanine residue at segment D3-S6 in Nav1.4 voltage-gated Na+ channels is critical for pyrethroid action. Mol Pharmacol. 2001;60(3):620–8. 11502895

[21] Soderlund DM, Knipple DC. The molecular biology of knockdown resistance to pyrethroid insecticides. Insect Biochem Molec. 2003;33(6):563–77. 10.1016/s0965-1748(03)00023-7 12770575

[22] Davies TGE, Williamson MS. Interactions of pyrethroids with the voltage-gated sodium channel. Bayer CropScience Journal. 2009;62:159–77.

[23] Barbosa S, Black WC, Hastings I. Challenges in Estimating Insecticide Selection Pressures from Mosquito Field Data. Plos Neglect Trop D. 2011;5(11). 10.1371/journal.pntd.0001387 PMC3206009 22069506

